# The effect of parecoxib sodium on postoperative delirium in elderly patients with hip arthroplasty

**DOI:** 10.3389/fphar.2023.947982

**Published:** 2023-03-20

**Authors:** Jin-Huo Wang, Tong Liu, Yu Bai, Yong-Quan Chen, Ying-Hui Cui, Xin-Yue Gao, Jian-Rong Guo

**Affiliations:** ^1^ Department of Anesthesiology and Perioperative Medicine, Gongli Hospital, Naval Military Medical University, Shanghai, China; ^2^ Department of Anesthesiology, The First Affiliated Hospital of Wannan Medical College, Wuhu, China

**Keywords:** postoperative delirium, parecoxib sodium, inflammation, HO-1, anesthesiology

## Abstract

**Objective:** This study aimed to clarify the effect of parecoxib sodium on the occurrence of postoperative delirium and to investigate its possible mechanism.

**Methods:** A total of 80 patients who underwent elective hip arthroplasty in our hospital between December 2020 and December 2021 were selected and randomly divided into two groups: a parecoxib sodium group (group P, *n* = 40) and a control group (group C, *n* = 40). Patients in group P were intravenously injected with 40 mg of parecoxib sodium 30 min before anesthesia and at the end of the surgery. Patients in group C were intravenously injected with the same volume of normal saline at the same time points. The primary endpoint was the incidence of POD, and the secondary endpoints were the levels of inflammatory factors (tumor necrosis factor- α [TNF-α], interleukin [IL]-1β, IL-6, and IL-10), nerve injury-related factors (brain-derived neurotrophic factor [BDNF], S-100β protein, neuron-specific enolase [NSE], and neurofilament light chain [NfL]), and antioxidant factors (heme oxygenase-1 [HO-1]), as well as the Visual Analogue Scale (VAS) and Confusion Assessment Method-Chinese Reversion (CAM-CR) scores.

**Results:** The incidence of POD was 10% in group P and 27.5% in group C. Intergroup comparison revealed that the levels of TNF-α, IL-1β, S-100β, NfL, and NSE were lower, and BDNF was higher, in group P than in group C at each postoperative time point. The levels of IL-6 were lower, and the levels of IL-10 and HO-1 were higher, in group P than in group C at 1 h and 1 day postoperatively (*p* < 0.05). Three days after surgery, the differences in the levels of IL-6, IL-10, and HO-1 were not statistically significant between the two groups (*p* > 0.05). The VAS and CAM-CR scores were lower at each postoperative time point in group P than in group C (*p* < 0.05).

**Conclusion:** Parecoxib sodium could reduce postoperative pain, decrease the plasma levels of inflammatory and nerve injury-related factors, upregulate HO-1 levels, and reduce the incidence of POD. The results of this study suggest that parecoxib sodium may reduce the occurrence of POD through the effects of anti-inflammation, analgesia, and antioxidants.

## 1 Introduction

Postoperative delirium (POD) is a common postoperative complication in elderly patients, especially those of very advanced age. It not only seriously affects the perioperative recovery of these patients but may also lead to a poor long-term prognosis (Albanese et al., 2022). The incidence of POD varies by population, type of procedure, timing (emergency or elective surgery), delirium assessment tool, and even ward location within a hospital, with significant variation in the incidence of POD across reports. It has, however, been shown that the incidence of POD in elderly patients undergoing surgery for hip fractures is approximately 30% (Ahmed and Kuo, 2022).

The pathogenesis of POD is currently unclear, but with the deepening of research into pathophysiological mechanisms, it is believed that the occurrence of POD is the result of multiple mechanisms complementing and influencing each other. Among the many hypotheses concerning the occurrence of POD, the most widely accepted ones are those concerning neuroinflammatory and oxidative stress response mechanisms (Gamberale et al., 2021).

Currently, multi-component non-pharmacological interventions—such as preoperative correction of susceptibility factors, improvement of physiological function reserve, and avoidance of possible predisposing factors in patients with high risk—are recommended for the prevention and treatment of POD (Hughes et al., 2020). Less invasive surgical procedures, strengthened intraoperative monitoring and management, improved postoperative analgesia and environment, and the avoidance of too much anesthesia, large fluctuations in blood pressure (BP) and blood glucose, and too high or too low body temperature, are also recommended. Although many studies have sought the possibility of drug prevention for POD, this is still a controversial subject, and there is a lack of strong evidence for drug prevention (Swarbrick and Partridge, 2022). It has been shown that the prophylactic administration of dexmedetomidine may reduce the risk of POD (Qin et al., 2021), but the exact effect is not clear (Turan et al., 2020; Patel et al., 2022).

A recent study found that, after hip and knee arthroplasty, multivariate models showed a relatively lower incidence of POD in patients who were given non-steroidal anti-inflammatory drugs (NSAIDs) and cyclooxygenase-2 (COX-2) inhibitors (Memtsoudis et al., 2019). The COX-2 inhibitor parecoxib sodium produces antipyretic, analgesic, and anti-inflammatory effects by selectively inhibiting the activity of COX-2 and thus blocking the conversion of arachidonic acid into prostaglandins COX-2 (Kaduševičius, 2021). Studies have shown that parecoxib sodium exerts anti-inflammatory effects by inhibiting the pro-inflammatory factors interleukin (IL)-1β and IL-8 and upregulating anti-inflammatory factor IL-10 and inhibits oxidative stress by suppressing reactive oxygen species and nitric oxide (Li and Zheng, 2021; Zhang et al., 2021). Given that the occurrence of POD is related to oxidative stress and inflammatory response, and parecoxib sodium inhibits oxidative stress and inflammatory response, it can be speculated that parecoxib sodium may have a positive effect on reducing the occurrence of POD. However, whether the effect on POD is direct or indirect remains unclear. The present study therefore investigated the effect of parecoxib sodium on POD and the possible mechanisms in order to provide a clinical reference for the prevention and treatment of POD.

## 2 Materials and methods

### 2.1 Study subjects

A total of 80 elderly patients who underwent elective hip arthroplasty in our hospital between December 2020 and December 2021 were selected according to the inclusion and exclusion criteria.

Inclusion criteria: Patients aged ≥65 years, with a body mass index of 18–28 kg/m^2^ and American Society of Anesthesiologists (ASA) grade I–III, who were able to complete the relevant scales independently.

Exclusion criteria: 1) Patients with a previous history of psychiatric or neurological disorders; 2) patients on long-term psychotropic medications; 3) patients with severe hepatic impairment, with serum albumin < 25 g/L or a Child–Pugh score ≥10; 4) patients with a history of allergy to sulfonamides or other NSAIDs; 5) patients with a history of gastrointestinal hemorrhage or perforation with the application of NSAIDs; 6) patients with a preoperative Mini-Mental State Examination (MMSE) score ≤ 23.

The present study was approved by the Ethics Committee of Gongli Hospital Affiliated to the Naval Medical University, and signed informed consent was obtained from patients or their families.

### 2.2 Sample size estimation and grouping

According to the pretest, the incidence of POD in elderly patients after total hip arthroplasty was approximately 30%, and with the application of parecoxib sodium, the incidence of POD decreased to approximately 5%. Therefore, the two-tailed α of 0.05, the test power 1-β of 0.8, and the dropout rate of 10% were selected. The final sample size calculated by PASS software version 15.0 was 40 cases in each group, with a total of 80 cases. Patients were divided into two groups using the random number table method: a parecoxib sodium group (group P, *n* = 40) and a control group (group C, *n* = 40). Patients in group P were intravenously injected with 40 mg of parecoxib sodium 30 min before anesthesia induction and at the end of the surgery. Patients in group C were intravenously injected with the same volume of normal saline at the same time points.

### 2.3 Anesthesia methods

All patients fasted (food and water) and took no preoperative medication. After entering the operation room, an electrocardiogram, arterial partial pressure of oxygen, heart rate, and BP were routinely monitored. The upper extremity infusion access was established, and radial artery puncture and catheterization were performed under local anesthesia for arterial pressure and blood gas monitoring. The right internal jugular vein was punctured and catheterized for central venous pressure monitoring and infusion.

Anesthesia induction: A total of 0.05 mg/kg of midazolam, 1.0–2.0 mg/kg of propofol, 0.3 μg/kg of sufentanil, and 0.2 mg/kg of cisatracurium was used for intravenous induction. Volume-controlled ventilation was then conducted *via* tracheal intubation in order to maintain the end-tidal partial pressure of carbon dioxide at 30–40 mmHg.

Anesthesia maintenance: A total of 3–6 mg kg^−1^·h^−1^ of propofol, 0.1–0.2 μg kg^−1^·min^−1^ of remifentanil, and 1.0–2.0 μg kg^−1^·min^−1^ of cisatracurium was continuously infused for target control infusion with the bispectral index being maintained at 40–60.

All surgeries were performed by the same team of surgeons. After the operation, a uniform formula of intravenous self-controlled analgesia was conducted. After reaching the indication for extubation, the tracheal tube was removed, and the patient was sent back to the ward if they had no special observation results.

### 2.4 Scale scores

The MMSE scale was scored at 1 day preoperatively (T0). The visual analogue scale (VAS) was scored at 1 day preoperatively (T0), 12 h postoperatively (T3), 1 day postoperatively (T4), and 2 days postoperatively (T5). The Confusion Assessment Method-Chinese Revision (CAM-CR) was scored at 1 day preoperatively (T0), 1 day postoperatively (T4), 3 days postoperatively (T6), and 5 days postoperatively (T7). These scoring was performed by the same trained anesthesiologist, and we used a blinded approach, meaning that the anesthesiologist performing the scale assessment did not know what medications the patient was on.

### 2.5 Plasma sample collection and assay

A total of 3 ml of central venous blood was collected from the internal jugular vein 30 min before anesthesia (T_1_), 1 h preoperatively (T_2_), 1 day preoperatively (T_4_), and 3 days preoperatively (T_6_), then centrifuged at 3,000 r/min for 10 min at room temperature. The supernatant was extracted and stored in a refrigerator at –80 °C for storage and further detection. The plasma levels of inflammatory factors (tumor necrosis factor α [TNF-α], IL-1β, IL-6, and IL-10), nerve injury-related factors (brain-derived neurotrophic factor [BDNF], S-100β protein, neuron-specific enolase [NSE], and neurofilament light chain [NfL]), and antioxidant factors (heme oxygenase-1 [HO-1]) were measured by enzyme-linked immunosorbent assay.

### 2.6 Statistical analysis

The SPSS 26.0 software was adopted for data processing. The measurement data were expressed as means ± standard deviation ( $\bar{x}$ *± s*). Repeated-measures analysis of variance was used for intra-group comparisons, and an independent sample *t*-test was used for inter-group comparisons. The countable data were expressed as the number of cases (%), and the χ^2^ test was adopted. *p* < 0.05 was considered statistically significant.

## 3 Results

### 3.1 General characteristics

The differences between the two groups in gender distribution, age, body weight, operation duration, infusion volume, blood loss, urine output, and preoperative MMSE score were not statistically significant (*p* > 0.05; see Table 1).

**TABLE 1:** Comparison of general conditions (*n* = 40).

Admission characteristics	Group P	Group C	*t/x* ^*2*^	*P*
Gender, male/female	12/28	15/25	0.503	0.478
Age, year	74.86 ± 5.38	73.43 ± 6.44	0.258	0.797
BMI, kg/m^2^	21.65 ± 3.76	22.15 ± 4.68	0.966	0.337
Operation Time, min	121.75 ± 40.46	124.54 ± 35.49	0.218	0.828
Fluid Input, ml	1,589.99 ± 394.54	1,564.00 ± 382.40	0.299	0.766
Blood Loss, ml	300.75 ± 56.13	285.00 ± 57.96	1.235	0.221
Urine Volume, ml	375.00 ± 131.56	391.24 ± 145.82	0.523	0.602
ASA grade, I/II/III	3/32/5	4/34/2	2.281	0.320
MMSE scores	28.04 ± 1.45	27.93 ± 1.44	0.604	0.718
Hypertension	17	15	0.208	0.820
Diabetes	11	13	0.238	0.808

Values are mean ± SD, or number.

BMI, body mass index; MMSE, Mini-Mental State Examination.

### 3.2 Inflammatory factors

**FIGURE 1:**
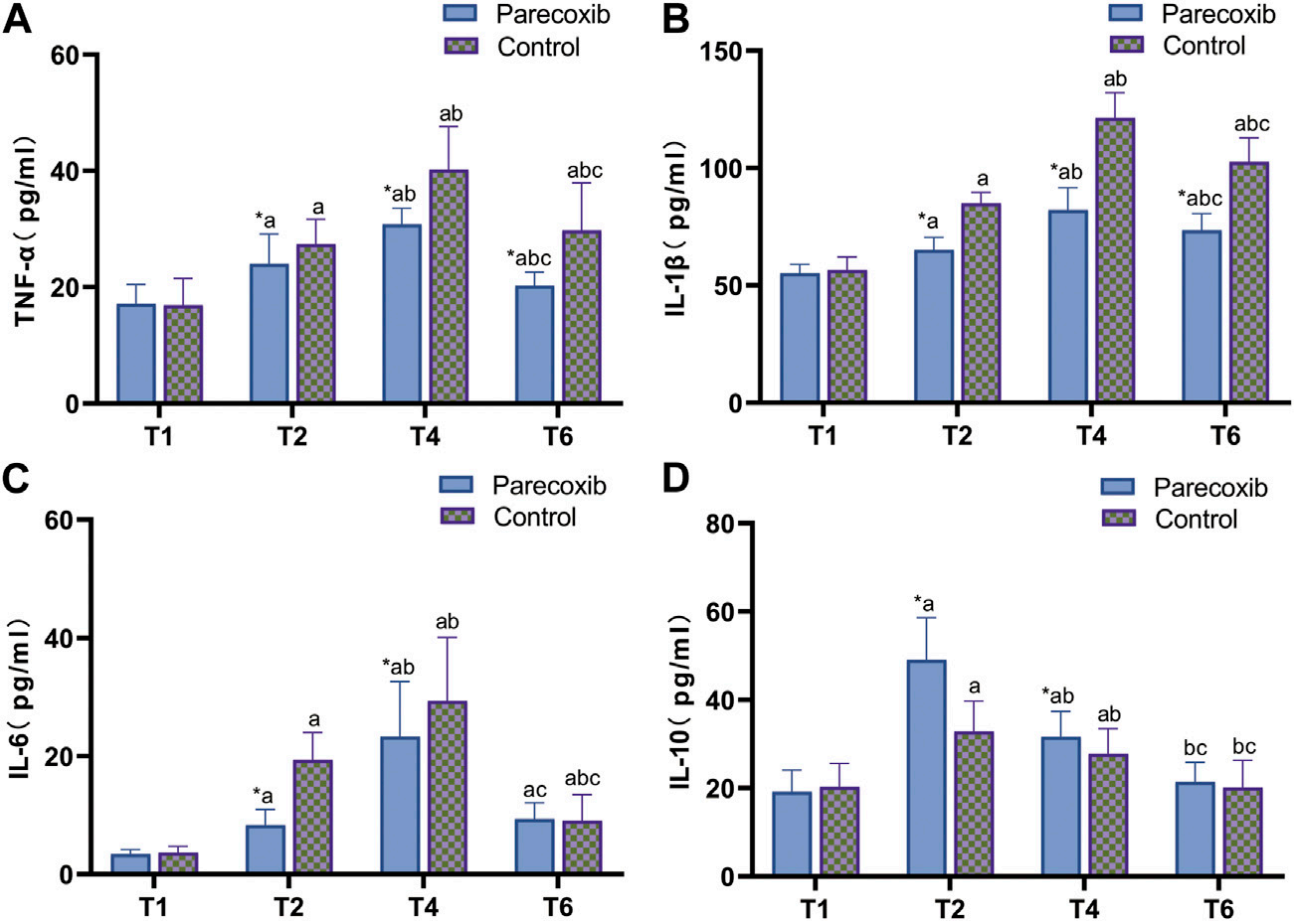
Changes of TNF-α **(A)**, IL-1β **(B)**, IL-6 **(C)** and IL-10 **(D)** at different time points.

Compared with those at T_1_, the levels of TNF-α, IL-1β, and IL-6 in the two groups were significantly increased at T_2_, T_4_, and T_6_ (*p* < 0.05), and the level of IL-10 was significantly increased at T_2_ and T_4_ (*p* < 0.05), but the changes were not statistically significant at T_6_ (*p* > 0.05). Compared with those at T_2_, the levels of TNF-α and IL-1β in the two groups were significantly increased at T_4_ and T_6_, the level of IL-10 was significantly decreased (*p* < 0.05), and the level of IL-6 was significantly increased at T_4_ in both groups (*p* < 0.05). At T_6_, the changes were not statistically significant in group P (*p* > 0.05), while the levels decreased significantly in group C (*p* < 0.05). Compared with those at T_4_, the levels of inflammatory factors in both groups were decreased at T_6_ (*p* < 0.05). Comparing between the two groups, there was no statistical significance of inflammatory factors in both groups at T_1_ time point (*p* > 0.05), TNF-α, IL-1β and IL-6 were lower in group P than group C and IL-10 was higher in group C at T_2_ and T_4_ time points (*p* < 0.05), TNF-α and IL-1β were lower in group P than group C at T_6_ time point (*p* < 0.05), and IL-6 and IL-10 were not statistically significant (*p* > 0.05) (*p* > 0.05; see Figure 1).

### 3.3 Nerve injury-related factors

**FIGURE 2:**
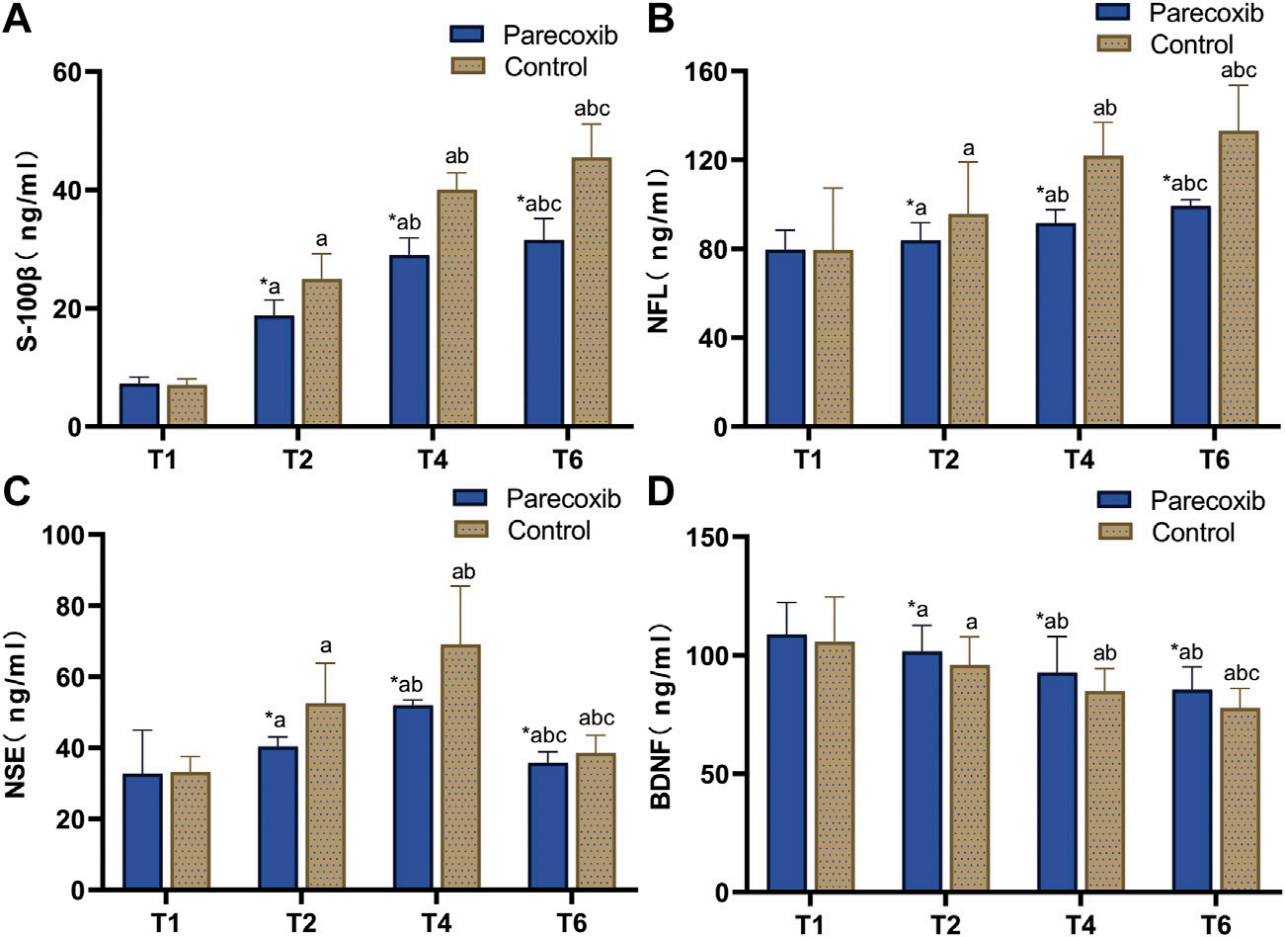
Changes of nerve injury related factors S-100β **(A)**, NFL **(B)**, NSE **(C)**, and BDNF **(D)** at different time points.

Compared with those at T_1_, S-100β, NFL, and NSE levels in the two groups at T_2_, T_4_, and T_6_ were significantly increased, while the levels of BDNF were significantly decreased (*p* < 0.05). Compared with those at T_2_, S-100β and NfL levels were significantly increased at T_4_ and T_6_, BDNF levels were significantly decreased, and NSE was significantly increased at T_4_ and decreased at T_6_ (*p* < 0.05). Compared with the T4 time point, at the T6 time point, S-100β and NFL were elevated and NSE was significantly lower in both groups (*p* < 0.05), BDNF was not significantly different in the P group (*p* > 0.05), and BDNF was significantly lower in the C group (*p* < 0.05) The results of the comparison between the two groups showed that there were no significant differences in nerve injury-related factors between the two groups at T_1_ (*p* > 0.05), while at T_2_, T_4_, and T_6_, S-100β, NfL, and NSE levels in group P were lower than those in group C, and BDNF levels were higher in group P than those in group C (*p* < 0.05; see Figure 2).

### 3.4 Antioxidant factors

Compared with those at T_1_, HO-1 levels in the two groups were significantly increased at T_2_ and T_4_ (*p* < 0.05), but the differences were not statistically significant at T_6_ (*p* > 0.05). Compared with those at T_2_, HO-1 levels in both groups were decreased at T_4_ and T_6_ (*p* < 0.05). Compared with those at T_4_, HO-1 was significantly decreased at T_6_ (*p* < 0.05). Intergroup comparison revealed that there was no significant difference in the level of HO-1 at T_1_ (*p* > 0.05), the levels of HO-1 at T_2_ and T_4_ in group P were higher than those in group C (*p* < 0.05), and the differences between the two groups at T_6_ were not statistically significant (*p* > 0.05; see Table 2).

**TABLE 2:** Changes of HO-1 expression levels at different time points ( $\bar{x}$ *± s*) , *n* = 40).

Index	Group	T_1_	T_2_	T_4_	T_6_
HO-1 (ng/ml)	P	41.96 ± 16.06	129.24 ± 43.35^a^ ^,^ ^b^	77.98 ± 14.22^a^ ^ab^	42.62 ± 14.13^bc^
	C	46.34 ± 12.58	81.81 ± 13.61^b^	71.43 ± 14.45^ab^	41.64 ± 12.45^bc^
	*t*	-1.358	6.601	2.042	0.331
	*P*	0.178	<0.001	0.044	0.742

^b^ *P* < 0.05, Compared with the T_1_.

^b^ *P* < 0.05, Compared with the T_2_.

^c^ *P* < 0.05, Compared with the T_4_.

^a^ *p* < 0.05, Compared with group C.

### 3.5 Scale scores

Compared with those at T_0_, the VAS scores at T_3_, T_4_, and T_5_ were significantly decreased (*p* < 0.05). Compared with those at T_3_, the VAS scores decreased at T_4_ and T_5_ (*p* < 0.05). Compared with those at T_4_, the VAS scores decreased significantly at T_5_ (*p* < 0.05). Intergroup comparison revealed that the difference between the two groups in VAS scores at T_1_ were not statistically significant (*p* > 0.05), while the VAS scores at T_3,_ T_4,_ and T_5_ were significantly lower in group P than those in group C (*p* < 0.05).

**FIGURE 3:**
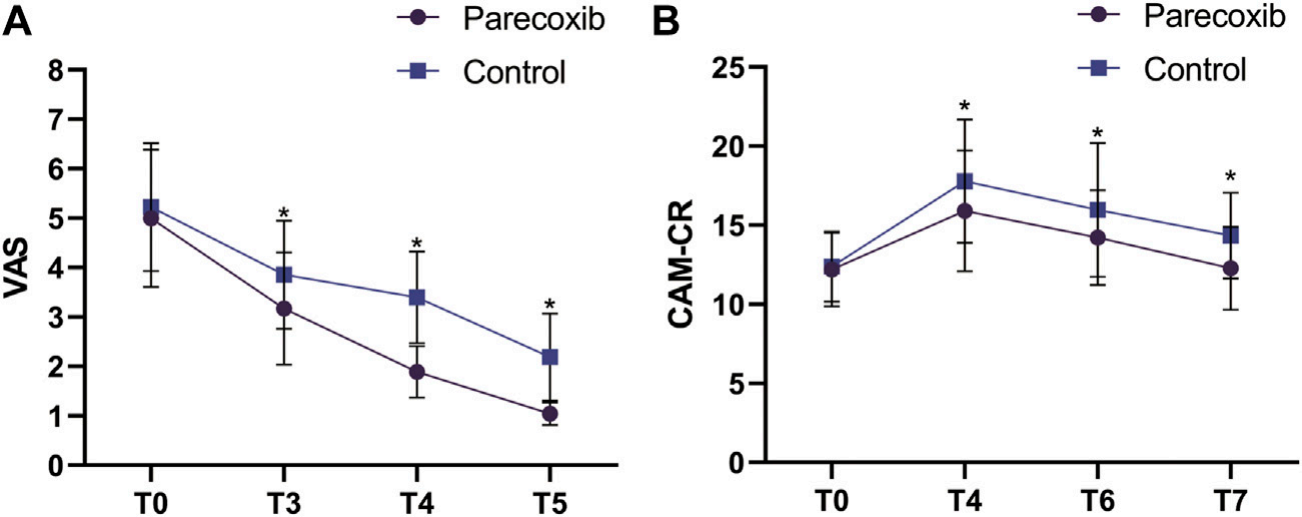
VAS **(A)** and CAM-CR **(B)** score changes at different time points.

Compared with those at T_0_, the CAM-CR scores at T_4_ and T_6_ were significantly increased (*p* < 0.05). The difference in CAM-CR scores at T_7_ in group P was not statistically significant (*p* > 0.05), while those in group C increased significantly (*p* < 0.05). Compared with those at T_4_, the CAM-CR scores decreased at T_6_ and T_7_ (*p* < 0.05). Compared with those at T_6_, the CAM-CR scores decreased significantly at T_7_ (*p* < 0.05). Intergroup comparison showed that there was no significant difference between the two groups in CAM-CR score at T_0_ (*p* > 0.05), and at T_4_, T_6_, and T_7_, the scores in group P were lower than those in group C (*p* < 0.05). The overall incidence of POD in group P was lower than that in group C (*p* < 0.05; see Figure 3 and Table 3).

**TABLE 3:** POD incidence at different time points in the two groups [n (%)].

Group	T_4_	T_6_	T_7_	Total incidence
P	4 (10%)	0 (0%)	0 (0%)	4 (10%)^a^
C	7 (17.5%)	3 (7.5%)	1 (2.5%)	11 (27.5%)

^a^ *p* < 0.05, Compared with group C.

## 4 Discussion

POD is a common neurological disorder in elderly patients during the perioperative period, with a peak occurrence of 24–72 h after surgery. It is characterized by impaired attention, disturbed consciousness, altered cognitive function, and marked fluctuations in the condition (Oh et al., 2017).

There are more factors affecting POD, and the study concluded that age, ASA classification; comorbidities and MMSE score were associated with the occurrence of POD (Mevorach et al., 2022), and in this study, age, ASA classification, MMSE score, length of surgery and comorbidities were not statistically different, ensuring the same between the two groups of patients at baseline.

Although the pathogenesis of POD remains unclear, it is currently thought to be the result of multiple mechanisms complementing and influencing each other. At present, the most widely accepted theories are those concerning neuroinflammatory response, neurotransmitter disorders, and oxidative stress (Gamberale et al., 2021). During surgical anesthesia, pathological processes like ischemia/reperfusion, hyperoxia, mitochondrial dysfunction, and hemolysis lead to oxidative stress in the body, and neuroinflammation due to oxidative stress and peripheral inflammation causes the release of large amounts of pro-inflammatory factors in the brain tissue, inducing the aggregation of inflammatory factors to produce an inflammatory response, which in turn leads to the development of neurological damage and the disruption of the expression levels of nerve injury-related factors.

The COX-2 inhibitor parecoxib sodium is an adjuvant for general anesthesia in surgery. It inhibits the aggregation of leukocytes, reduces the formation of bradykinin, inhibits platelet agglutination, and induces apoptosis and anti-angiogenesis by selectively inhibiting the activity of COX-2, thus producing antipyretic, analgesic, anti-inflammatory, and anti-cancer effects (Kaduševičius, 2021). Studies have shown that in major surgeries such as thoracic surgery and orthopedics, parecoxib sodium can reduce pain levels and postoperative serum inflammatory factor levels, reduce the incidence of adverse reactions such as nausea and vomiting and pulmonary infections, reduce analgesic drug consumption, reduce hemodynamic instability, and reduce surgery-related stress reactions (Yang et al., 2020; Li et al., 2022a). Basic research has revealed that parecoxib sodium can exert neuroprotective effects by inhibiting and improving mitochondrial dysfunction in the substantia nigra *via* the COX-2/PGE2 pathway (Yan et al., 2021). Parecoxib sodium also downregulates reactive oxygen species and lipid peroxidation levels, thereby attenuating oxidative stress and regulating redox imbalance by modulating NF-κB and Nrf-2 (Wu et al., 2021).

Since the occurrence of POD is associated with oxidative stress and inflammatory response, and parecoxib sodium has the effect of inhibiting oxidative stress and inflammatory response, it can be speculated that parecoxib sodium may have a positive effect on reducing the occurrence of POD.

Usually, inflammation can be observed by detecting changes in the levels of inflammatory mediators. A meta-analysis found that inflammatory mediators associated with POD include IL- 6, IL-8, IL-10, TNF-α, C-reactive protein, insulin-like growth factor-1, cortisol, and neopterin (Noah et al., 2021). Of these, increased pro-inflammatory factors, such as TNF-α, IL-1β, and IL-6, were positively correlated with the development of POD (Liu et al., 2021). IL-6 has been found to be a significant predictor of POD in various surgical conditions (elective surgery or emergency) (Adamis et al., 2021). It has also been shown that decreasing the expression of IL-6 and upregulating the expression of IL-10 in the peripheral blood, hippocampus, and prefrontal cortex reduces microglia activation and reduces delirium-like behavior in mice (Li et al., 2022b).

HO-1 is an important antioxidant mediator in the body and is involved in protecting several tissues and organs from oxidative stress and excessive inflammatory responses by releasing a variety of molecules with antioxidant stress and antioxidant effects (Yachie, 2021). Levels of HO-1 are positively correlated with the degree of oxidative stress response. In the present study, the expression levels of TNF-α, IL-1β, and IL-6 in group P were lower than those in group C at all postoperative time points, and the expression levels of IL-10 were higher than those in group C. The level of HO-1 in group P was significantly higher than in group C at 1 h postoperatively but returned to the preoperative level at 3 days after surgery. These results suggest that parecoxib sodium might inhibit pro-inflammatory factors, upregulate anti-inflammatory factors, and promote the early expression of HO-1, thereby reducing the inflammation cascade in peripheral monocytes and the activation of microglia and astrocytes in the central nervous system, decreasing the degree of neuroinflammatory injury and blocking the damage caused by oxidative stress pathways at an early stage.

S-100β has been found to be significantly elevated in the serum of patients who have experienced or are experiencing delirium (Khan et al., 2021), suggesting that there might be a close relationship between delirium and nerve injury and that factors such as surgery, hemorrhage, anesthetic drugs, and perioperative hypoperfusion might cause cerebral injury and contribute to the development of delirium. It has been reported that S-100β in the blood is positively correlated with the occurrence of POD (Wang et al., 2022). Axons release small amounts of NfL—an important component subunit of neuronal neurofilament proteins—under normal physiological conditions, but under pathological conditions, the synthesis and release of NfL increases significantly. Although NfL can be exchanged among tissue fluid, cerebrospinal fluid, and blood, the concentration of NfL in the blood is still lower than that in the cerebrospinal fluid. The concentrations of NfL in cerebrospinal fluid and blood are strongly correlated with the degree of neuropathy, so NfL is adopted clinically as a biomarker for diseases associated with neural injury. A positive correlation has also been found between the level of NfL in serum and that in cerebrospinal fluid, and it has been found that patients with high NfL levels are more likely to develop POD (Mietani et al., 2021). Elevated NSE levels are also associated with POD, and early monitoring of serum NSE levels, as well as assessment in conjunction with clinical manifestations, may improve diagnostic accuracy for POD (Fong et al., 2020). BDNF mainly affects neuroplasticity and neurotransmission, and the reduced level increases the incidence of delirium (Xiang et al., 2021). In the present study, the nerve injury-related factors S-100β and NSE were lower in group P than in group C at all time points, and the levels of BDNF were higher in group P than in group C. These findings indicate that parecoxib sodium might reduce the production of nerve injury-related factors, increase the level of protective neural factors, and reduce the degree of neural injury.

Severe pain, both preoperative and postoperative, may increase stress and the occurrence of POD in patients (Ding et al., 2021; Liu et al., 2022). The higher the degree of pain, the higher the incidence of POD, which might be related to the fact that pain can significantly affect sleep and disrupt the sleep-wake cycle in the patient, thus impairing cognitive function (Jaiswal et al., 2020). In addition, high-dose opioid use has been associated with an increased incidence of POD (Zhu et al., 2020). In previous studies, parecoxib sodium provided good analgesia and reduced opioid use, while also reducing opioid-induced nociceptive hypersensitivity (Kellett et al., 2021; Lyu et al., 2022). In the present study, the VAS scores at 12 h, 24 h, and 48 h postoperatively in group P were lower than those in group C, indicating the significant analgesic effect of parecoxib sodium postoperatively. This suggests that parecoxib sodium could be used to reduce the interference of pain with sleep rhythms, thereby contributing to the decreased incidence of POD.

The Confusion Assessment Method (CAM) is currently the most commonly used tool for assessing POD in elderly postoperative patients, and it is suitable for application by non-psychiatrists (Ho et al., 2021). However, there were some problems in its clinical application in China. Therefore, the CAM-CR was developed according to the clinical situation in China, making the quantitative assessment of the occurrence and status of delirium easier (Li et al., 2003). In the present study, the CAM-CR scores and overall incidence of POD at all postoperative time points were lower in group P than in group C, suggesting that parecoxib sodium can reduce the CAM-CR score and the incidence of POD, which might be related to the effects of analgesia, anti-inflammation, and antioxidative stress.

## 5 Conclusion

The COX-2 inhibitor parecoxib sodium may reduce postoperative pain, decrease plasma levels of inflammatory factors and nerve injury-related factors after surgery, upregulate IL-10, BDNF, and HO-1 levels, and reduce the overall incidence of postoperative POD in elderly patients. The results of this study suggest that parecoxib sodium might improve the occurrence of POD by reducing neuroinflammation and increasing antioxidant effects. For patients at high risk of POD, a single dose of parecoxib sodium can be administered 30 min before surgery to reduce the incidence of POD. The limitations of this study are that this study is a single-center small sample trial and needs to be further confirmed in a multi-center large sample trial. In addition, the study was conducted on a single disease type, and further studies are needed to determine whether the same effect can be achieved in other diseases.

## Data Availability

The original contributions presented in the study are included in the article/supplementary material, further inquiries can be directed to the corresponding authors.

## References

[B1] Adamis D. van Gool W. A. Eikelenboom P. (2021). Consistent patterns in the inconsistent associations of Insulin-like growth factor 1 (IGF-1), C-Reactive Protein (C-RP) and Interleukin 6 (IL-6) levels with delirium in surgical populations. A systematic review and meta-analysis. Arch. Gerontol. Geriatr. 97, 104518. 10.1016/j.archger.2021.104518 34536657

[B2] Ahmed N. Kuo Y. H. (2022). Delirium risk in geriatric hip hemi-arthroplasty (DRIGHA): Development and validation of a novel score using a national data. Injury 53 (4), 1469–1476. 10.1016/j.injury.2022.01.041 35144810

[B3] Albanese A. M. Ramazani N. Greene N. Bruse L. (2022). Review of postoperative delirium in geriatric patients after hip fracture treatment. Geriatr. Orthop. Surg. Rehabil. 13, 21514593211058947. 10.1177/21514593211058947 35282299 PMC8915233

[B4] Ding X. Gao X. Chen Q. Jiang X. Li Y. Xu J. (2021). Preoperative acute pain is associated with postoperative delirium. Pain Med. 22 (1), 15–21. 10.1093/pm/pnaa314 33040141

[B5] Fong T. G. Vasunilashorn S. M. Ngo L. Libermann T. A. Dillon S. T. Schmitt E. M. (2020). Association of plasma neurofilament light with postoperative delirium. Ann. Neurol. 88 (5), 984–994. 10.1002/ana.25889 32881052 PMC7581557

[B6] Gamberale R. D'Orlando C. Brunelli S. Meneveri R. Mazzola P. Foti G. (2021). Study protocol: Understanding the pathophysiologic mechanisms underlying delirium in older people undergoing hip fracture surgery. BMC Geriatr. 21 (1), 633. 10.1186/s12877-021-02584-1 34736422 PMC8567587

[B7] Ho M. H. Nealon J. Igwe E. Traynor V. Chang H. C. R. Chen K. H. (2021). Postoperative delirium in older patients: A systematic review of assessment and incidence of postoperative delirium. Worldviews Evid. Based Nurs. 18 (5), 290–301. Epub 2021 Sep 4. PMID: 34482593. 10.1111/wvn.12536 34482593

[B8] Hughes C. G. Boncyk C. S. Culley D. J. Fleisher L. A. Leung J. M. McDonagh D. L. (2020). American society for enhanced recovery and perioperative quality initiative joint consensus statement on postoperative delirium prevention. Anesth. Analg. 130 (6), 1572–1590. 10.1213/ANE.0000000000004641 32022748 PMC7379173

[B9] Jaiswal S. J. Kang D. Y. Wineinger N. E. Owens R. L. (2020). Objectively measured sleep fragmentation is associated with incident delirium in older hospitalized patients: Analysis of data collected from an randomized controlled trial. J. Sleep. Res. 30, e13205. 10.1111/jsr.13205 33051948

[B10] Kaduševičius E. (2021). Novel applications of NSAIDs: Insight and future perspectives in cardiovascular, neurodegenerative, diabetes and cancer disease therapy. Int. J. Mol. Sci. 22 (12), 6637. 10.3390/ijms22126637 34205719 PMC8235426

[B11] Kellett E. Berman R. Morgan H. Collins J. (2021). Parecoxib for opioid-induced hyperalgesia. BMJ Support Palliat. Care 11 (2), 126–127. 10.1136/bmjspcare-2020-002290 32601152

[B12] Khan S. H. Lindroth H. Jawed Y. Wang S. Nasser J. Seyffert S. (2021). Serum biomarkers in postoperative delirium after esophagectomy. Ann. Thorac. Surg. 113, 1000. 10.1016/j.athoracsur.2021.03.035 33774004 PMC8582321

[B13] Li J. Zou Y. Z. Feng F. (2003). Reversion of CAM for assisting the evaluation and diagnosis of delirium (in Chinese). J. Clin. Psychiatry 2003 (03), 147–149.

[B14] Li K. Wang J. Chen L. Guo M. Zhou Y. Li X. (2022). Netrin-1 ameliorates postoperative delirium-like behavior in aged mice by suppressing neuroinflammation and restoring impaired blood-brain barrier permeability. Front. Mol. Neurosci. 14, 751570. 10.3389/fnmol.2021.751570 35095412 PMC8797926

[B15] Li M. Zheng Z. (2021). Protective effect of parecoxib sodium against ischemia reperfusion-induced intestinal injury. Mol. Med. Rep. 24 (5), 776. 10.3892/mmr.2021.12416 34498709 PMC8436217

[B16] Li X. Zhou P. Li Z. Tang H. Zhai S. (2022). Intravenous parecoxib for pain relief after orthopedic surgery: A systematic review and meta-analysis. Pain Ther. 11 (3), 771–787. 10.1007/s40122-022-00400-1 35705843 PMC9314469

[B17] Liu J. Shen Q. Zhang H. Xiao X. Lv C. Chu Y. (2021). The potential protective effect of mesencephalic astrocyte-derived neurotrophic factor on post-operative delirium via inhibiting inflammation and microglia activation. J. Inflamm. Res. 14, 2781–2791. 10.2147/JIR.S316560 34234505 PMC8254188

[B18] Liu Y. M. Huang H. Gao J. Zhou J. Chu H. C. (2022). Hemoglobin concentration and post-operative delirium in elderly patients undergoing femoral neck fracture surgery. Front. Med. (Lausanne). 8, 780196. 10.3389/fmed.2021.780196 35071265 PMC8766508

[B19] Lyu N. Kong Y. Li X. Guo N. Lai J. Li J. (2022). Effect and safety of prophylactic parecoxib for pain control of transarterial chemoembolization in liver cancer: A single-center, parallel-group, randomized trial. J. Am. Coll. Radiol. 19 (1), 61–70. 10.1016/j.jacr.2021.09.029 34736908

[B20] Memtsoudis S. Cozowicz C. Zubizarreta N. Weinstein S. M. Liu J. Kim D. H. (2019). Risk factors for postoperative delirium in patients undergoing lower extremity joint arthroplasty: A retrospective population-based cohort study. Reg. Anesth. Pain Med. 44, 934–943. rapm-2019-100700. 10.1136/rapm-2019-100700 31302641

[B21] Mevorach L. Forookhi A. Farcomeni A. Romagnoli S. Bilotta F. (2022). Perioperative risk factors associated with increased incidence of postoperative delirium: Systematic review, meta-analysis, and grading of recommendations assessment, development, and evaluation system report of clinical literature. Br. J. Anaesth. 130 (22), e254. 10.1016/j.bja.2022.05.032 35810005

[B22] Mietani K. Hasegawa-Moriyama M. Inoue R. Ogata T. Shimojo N. Kurano M. (2021). Elevated neuron-specific enolase level is associated with postoperative delirium and detection of phosphorylated neurofilament heavy subunit: A prospective observational study. PLoS One 16 (11), e0259217. 10.1371/journal.pone.0259217 34797829 PMC8604326

[B23] Noah A. M. Almghairbi D. Evley R. Moppett I. K. (2021). Preoperative inflammatory mediators and postoperative delirium: Systematic review and meta-analysis. Br. J. Anaesth. 127 (3), 424–434. 10.1016/j.bja.2021.04.033 34218905

[B24] Oh E. S. Fong T. G. Hshieh T. T. Inouye S. K. (2017). Delirium in older persons: Advances in diagnosis and treatment. JAMA 318 (12), 1161–1174. 10.1001/jama.2017.12067 28973626 PMC5717753

[B25] Patel M. Onwochei D. N. Desai N. (2022). Influence of perioperative dexmedetomidine on the incidence of postoperative delirium in adult patients undergoing cardiac surgery. Br. J. Anaesth. 129, 67–83. (21)00859-X. 10.1016/j.bja.2021.11.041 35279278

[B26] Qin C. Jiang Y. Lin C. Li A. Liu J. (2021). Perioperative dexmedetomidine administration to prevent delirium in adults after non-cardiac surgery: A systematic review and meta-analysis. J. Clin. Anesth. 73, 110308. 10.1016/j.jclinane.2021.110308 33930679

[B27] Swarbrick C. J. Partridge J. S. L. (2022). Evidence-based strategies to reduce the incidence of postoperative delirium: A narrative review. Anaesthesia 77 (1), 92–101. 10.1111/anae.15607 35001376

[B28] Turan A. Duncan A. Leung S. Karimi N. Fang J. Mao G. (2020). Dexmedetomidine for reduction of atrial fibrillation and delirium after cardiac surgery (DECADE): A randomised placebo-controlled trial. Lancet 396 (10245), 177–185. 10.1016/S0140-6736(20)30631-0 32682483

[B29] Wang S. Greene R. Song Y. Chan C. Lindroth H. Khan S. (2022). Postoperative delirium and its relationship with biomarkers for dementia: A meta-analysis. Int. Psychogeriatr. 34, 377–390. 10.1017/S104161022100274X PMC9288560 35034675

[B30] Wu F. Wang W. Duan Y. Guo J. Li G. Ma T. (2021). Effect of parecoxib sodium on myocardial ischemia-reperfusion injury rats. Med. Sci. Monit. 27, e928205. 10.12659/MSM.928205 33395402 PMC7791896

[B31] Xiang X. B. Chen H. Wu Y. L. Wang K. Yue X. Cheng X. Q. (2021). The effect of pre-operative methylprednisolone on postoperative delirium in elderly patients undergoing gastrointestinal surgery: A randomized, double-blind, placebo-controlled trial. J. Gerontol. A Biol. Sci. Med. Sci. 77, 517. 10.1093/gerona/glab248 34423832

[B32] Yachie A. (2021). Heme oxygenase-1 deficiency and oxidative stress: A review of 9 independent human cases and animal models. Int. J. Mol. Sci. 22 (4), 1514. 10.3390/ijms22041514 33546372 PMC7913498

[B33] Yan H. Zhao H. Kang Y. Ji X. Zhang T. Wang Y. (2021). Parecoxib alleviates the motor behavioral decline of aged rats by ameliorating mitochondrial dysfunction in the substantia nigra via COX-2/PGE2 pathway inhibition. Neuropharmacology 194, 108627. 10.1016/j.neuropharm.2021.108627 34089729

[B34] Yang J. Hao Z. Li W. Duan C. Fan X. Xin J. (2020). The efficacy and safety of paravertebral block combined with parecoxib during video-assisted thoracic surgery: A randomized controlled trial. J. Pain Res. 13, 355–366. 10.2147/JPR.S244787 32104057 PMC7025740

[B35] Zhang C. Hu S. Zosky G. R. Wei X. Shu S. Wang D. (2021). Paracoxib alleviates ventilator-induced lung injury through functional modulation of lung-recruited CD11bloLy6Chi monocytes. Shock 55 (2), 236–243. PMID: 32590697. 10.1097/SHK.0000000000001591 32590697

[B36] Zhu C. Wang B. Yin J. Xue Q. Gao S. Xing L. (2020). Risk factors for postoperative delirium after spinal surgery: A systematic review and meta-analysis. Aging Clin. Exp. Res. 32 (8), 1417–1434. 10.1007/s40520-019-01319-y 31471892

